# Transcriptomic Analysis of Early Fruit Development in Micro-Tom Tomato Reveals Conserved and Cultivar-Specific Mechanisms

**DOI:** 10.3390/plants15010137

**Published:** 2026-01-03

**Authors:** Pedro Boscariol Ferreira, Simara Larissa Fanalli, Perla Novais de Oliveira, Aline da Silva Mello Cesar, Nubia Barbosa Eloy

**Affiliations:** 1 Departamento de Ciências Biolóogicas, Escola Superior de Agricultura ‘Luiz de Queiroz’ (ESALQ), University of São Paulo (USP), Piracicaba 13418-900, Brazil; perla.oliveira@usp.br; 2 Departamento de Ciência e Tecnologia de Alimentos, Escola Superior de Agricultura ‘Luiz de Queiroz’ (ESALQ), University of São Paulo (USP), Piracicaba 13418-900, Brazil; simarafanalli@usp.br (S.L.F.); alinecesar@usp.br (A.d.S.M.C.)

**Keywords:** Micro-Tom tomato, fruit development, transcriptomics, genome assembly, gene expression, protein interaction networks

## Abstract

Early fruit development in tomato is driven by complex gene expression patterns and metabolic reprogramming, a crucial phase that shapes the fruit’s final size and structure. Previous studies using the Micro-Tom model have largely focused on later stages of development, especially ripening, leaving early developmental processes relatively unexplored. To address this knowledge gap, we performed RNA-seq analyses on Micro-Tom fruits harvested at three key developmental stages: 3, 5, and 8 days post-anthesis (DPA). Pairwise differential gene expression analyses revealed that the most extensive transcriptional reprogramming occurs during the transition from 5 to 8 DPA, while comparatively fewer changes were observed between 3 and 5 DPA. K-means clustering of 11,035 stably expressed genes revealed nine distinct expression profiles associated with specific developmental phases, including cell proliferation, transition, and cell expansion. Integrating transcriptomic and metabolomic datasets uncovered coordinated shifts in gene expression and metabolite accumulation, highlighting both conserved regulatory mechanisms and cultivar-specific pathways governing early fruit development. These findings advance our understanding of the molecular regulation of early fruit development in Micro-Tom tomatoes and provide a basis for future efforts to improve fruit quality and yield.

## 1. Introduction

Tomato (*Solanum lycopersicum*) is an important model species for studying fleshy fruit development due to its extensive phenotypic diversity, particularly in fruit weight and morphology [1]. This diversity provides valuable insights into the fundamental biological processes of fruit growth, ripening, and hormonal regulation. Fruit development in tomato encompass a biphasic pattern including an initial phase of active cell division post-anthesis, followed by a phase of cell expansion without clear boundaries between these stages [2,3]. Hormones, notably gibberellins (GAs) and auxins, play critical roles in controlling fruit set and development. Gibberellins promote cell division, while auxins primarily promote cell expansion, influencing overall fruit size and morphogenesis [4].

The early developmental stages are critical for fruit formation and final fruit size. Shortly after anthesis, fertilization determines whether fruit development will continue or abort, a process known as fruit set [5,6]. The size and weight of the fruit largely depend on the number of cells in the pericarp, established during the cell division stage [7]. Following fertilization, the embryo within the ovule undergoes rapid proliferation, reaching a multicellular embryonic stage around 3 to 6 days post-anthesis (DPA) [8]. Simultaneously, cells in the ovary wall begin dividing around 2 DPA, with cell division in the ovary wall nearly completed by 5 DPA, marking the onset of the cell expansion phase [8,9,10].

Several genes have been identified that affect fruit size by regulating cell division during these early developmental stages. Despite the wide diversity in fruit size and shape observed across cultivated tomatoes, a limited set of genes seems to drive the major variations in fruit morphology among domesticated varieties [11,12]. Alleles of the major genes *SUN*, *OVATE*, *LOCULE NUMBER*, and *FASCIATED* individually explain up to 71% of the fruit shape variations in cultivated tomato [13]. Quantitative trait loci-associated genes *Cell Number Regulator* and *SlKLUH* (from QTLs *fw2.2* and *fw3.2*, respectively) also influence fruit size through modulation of cell division rates [14].

The dwarf tomato cultivar Micro-Tom has emerged as a popular model system for genetic and developmental studies, due to its compact size (~15 cm in height), short life cycle (~70 days from germination to fruiting), and adaptability to high-density cultivation [15,16]. Originally bred as an ornamental variety, Micro-Tom carries distinctive mutations, including the *dwarf* (*d*) and *self-pruning* (*sp*) alleles, that underlie its reduced size and determine growth habit [16]. These traits, along with the *uniform ripening* (*u*) gene, make it an ideal controlled experimental model, particularly for studying plant hormone interactions and responses to environmental stimuli [17]. The uniform ripening phenotype is especially advantageous for fruit development research, as it enables synchronized sampling of large quantities of fruit tissue for downstream analyses. Furthermore, Micro-Tom possesses a relatively compact diploid genome of approximately 900 Mb and is supported by extensive genetic and genomic resources, which have collectively facilitated major advances in elucidating the molecular and genetic regulation of fruit formation [18,19].

Transcriptomic studies in tomato, particularly those using the Micro-Tom model, have provided valuable insights into gene expression and regulatory networks throughout different developmental stages. Tissue-specific transcriptome profiles have been mapped across essential reproductive organs, shedding light on the dynamics of gene regulation influencing fruit formation and maturation [20,21]. These studies have highlighted the complexity of gene regulatory networks driving the transition from ovary development to fruit maturation, including the roles of specific loci such as *SUN* and *OVATE* in determining fruit shape and the processes leading cellular expansion and endoreduplication [1].

More recently, cutting-edge approaches such as spatial transcriptomics [5,14] have begun to uncover the cellular heterogeneity and spatial organization of gene expression during fruit development. However, most studies focus on fruit ripening [22,23,24] and stress responses [25], with fewer transcriptomic analyses of early fruit development. An RNA-seq-based investigation of early tomato fruit development was performed in the ‘Moneymaker’ cultivar [14], providing insights into gene expression dynamics during the initial stages of fruit formation. This study identified key regulators involved in pericarp- and ovule-specific development post-fertilization, highlighting the roles of auxin biosynthesis genes and tissue-specific transcription factors. Nonetheless, comprehensive transcriptomic analyses focusing on the early stages of fruit development in the Micro-Tom cultivar remain lacking.

In this study, we used the most recent, high-quality assembly of the Micro-Tom tomato genome [18,19] to investigate organ-level transcriptomic changes associated with early fruit development. We selected three critical time points: 3 DPA, representing the cell division stage; 5 DPA, corresponding to the peak of cell proliferation; and 8 DPA, marking the transition to cell expansion [26]. At 3–4 DPA the pericarp begins adding layers as cell division proceeds; by 7–8 DPA, cell expansion becomes apparent [8,27]. During these stages, fruits comprise the pericarp (exocarp, mesocarp, and endocarp), the placenta and locules, and developing seeds [27]. This sampling window captures the shift from division-driven to expansion-driven programs without implying tissue specificity. Focusing on predicted protein-coding genes, we identified nine distinct expression clusters and annotated them through functional enrichment analysis to highlight the biological processes associated with each developmental stage. Additionally, integration of previously published GC-MS and LC-MS analyses in the same stages provided complementary context, revealing concordance between transcript abundance and metabolite profiles during this critical developmental window.

## 2. Results

### 2.1. RNA-Seq Quality and Alignment Statistics

RNA-seq statistics indicate high-quality data suitable for downstream analysis. On average, 80.7 ± 4.2% of raw reads passed quality filtering and trimming, resulting in read libraries with a mean Q30 of 94.86 ± 0.17% (Table 1). These libraries were successfully aligned to the Micro-Tom reference genome [19] with an average of 91.8 ± 0.9% of reads mapping uniquely (Table 1). Gene body coverage analysis shows a characteristic bell-shaped distribution, typical of high-quality poly(A)-selected libraries [28] and does not indicate degradation or technical artifacts (Supplementary Figure S1). In addition, Principal Component Analysis (PCA) before and after Trimmed Mean of M-value (TMM) normalization show consistent clustering by developmental stage, with normalization having a minimal impact on overall sample relationships (Supplementary Figure S2).

### 2.2. Differential Expression Shows Stage-Specific Transcriptional Changes

**Figure 1:**
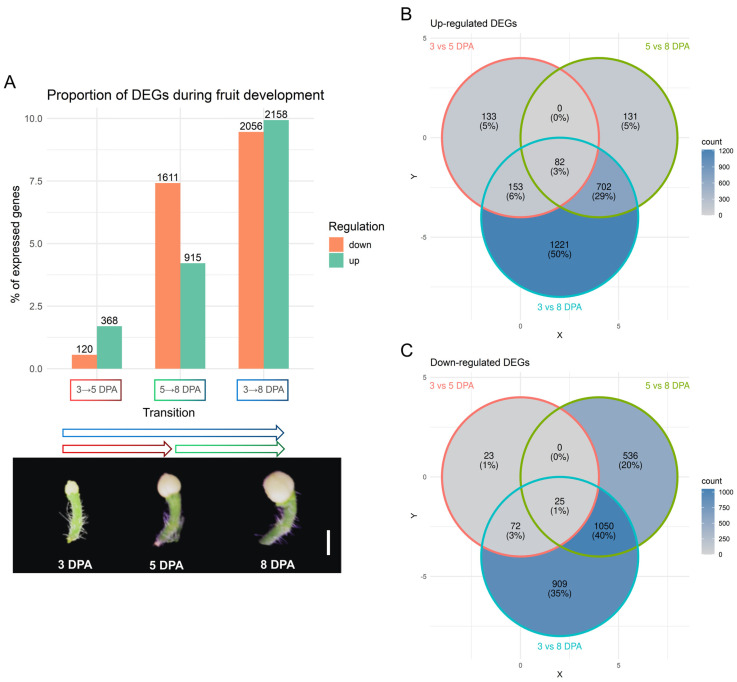
Description of differential gene expression. (**A**) Number of significant up- and down-regulated genes and their percentage in relation to all expressed genes in the dataset (21,725) in each transition between the fruit stages depicted below, bar = 0.5 cm; (**B**) Venn Diagram of significant up-regulated genes between comparisons; (**C**) Venn Diagram of significant down-regulated genes between comparisons.

To characterize gene expression dynamics during early fruit development, we performed pairwise differential expression analysis between 3 DPA vs. 5 DPA, 5 DPA vs. 8 DPA, and 3 DPA vs. 8 DPA (Figure 1A). Applying a threshold of |log2FC| > 1.5 and FDR < 0.01, we identified 368 upregulated and 120 downregulated genes between 3 and 5 DPA, 915 upregulated and 1611 downregulated genes between 5 and 8 DPA, and 2158 upregulated and 2056 downregulated genes across the 3 to 8 DPA interval (Figure 1A). These differentially expressed genes (DEGs) represent 2.25%, 11.63%, and 19.4% of all genes expressed in the dataset, respectively, based on a total of 21,725 genes retained after filtering for low expression.

Venn diagram analyses of significant DEGs show high overlap between the 5 vs. 8 DPA comparison and the 3 vs. 8 DPA set: 702 upregulated and 1050 downregulated genes are shared (Figure 1B,C). The 5 vs. 8 DPA comparison reveals the most pronounced transcriptomic shift, while the 3 vs. 5 DPA show a more modest change. The 3 vs. 8 DPA comparison largely reflects cumulative differences that emerge at the later stages (Figure 1).

Gene Ontology (GO) enrichment analysis of Biological Process terms show that between 3 and 5 DPA (Supplementary Figure S3), upregulated genes are enriched in processes such as response to wounding, fatty acid biosynthesis, and cellulose production (complete results in Supplementary File S1). No strongly enriched downregulated terms were detected at this stage.

In the 5 to 8 DPA transition (Supplementary Figure S4), upregulated genes were strongly associated with lipid storage, methylation, and pectin catabolism. Concurrently, downregulated genes were enriched in defense responses, microtubule-based movement, and carbohydrate metabolism.

Across the 3 to 8 DPA interval (Supplementary Figure S5), upregulated genes were predominantly enriched for photosynthesis, chloroplast biogenesis, and fatty acid biosynthesis. In contrast, downregulated genes were associated with protein phosphorylation, defense response to fungi, and cell wall modification.

### 2.3. Distinct Expression Clusters Mark Developmental Transitions

**Figure 2:**
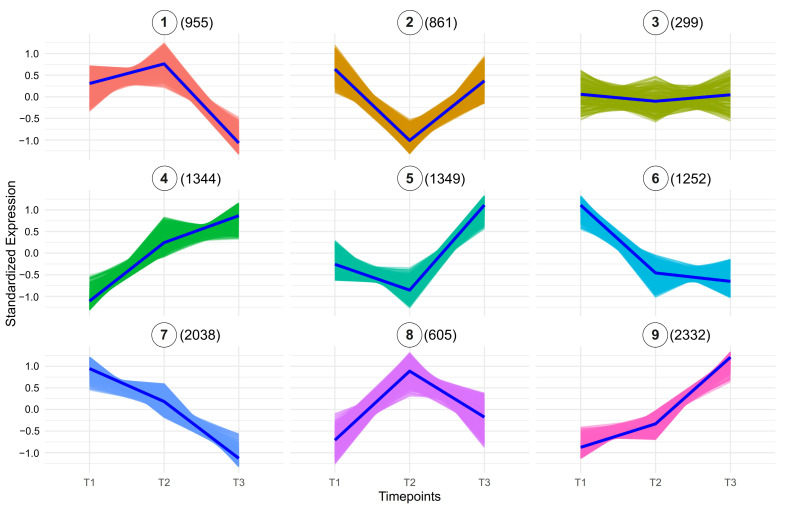
Expression clustering analysis of genes during three developmental stages (3 DPA, 5 DPA, and 8 DPA). Clusters are labeled from Cluster 1 through Cluster 9. Each panel represents a unique cluster, with the average scaled counts at the y-axis (TMM normalization followed by log_2_(x + 1) transformation and Z-score scaling) across the time points (x-axis). The number of genes within each cluster is shown in parentheses next to the cluster number. Each timepoint comprises three biological replicates. T1 = 3 DPA, T2 = 5 DPA, and T3 = 8 DPA.

**Figure 3:**
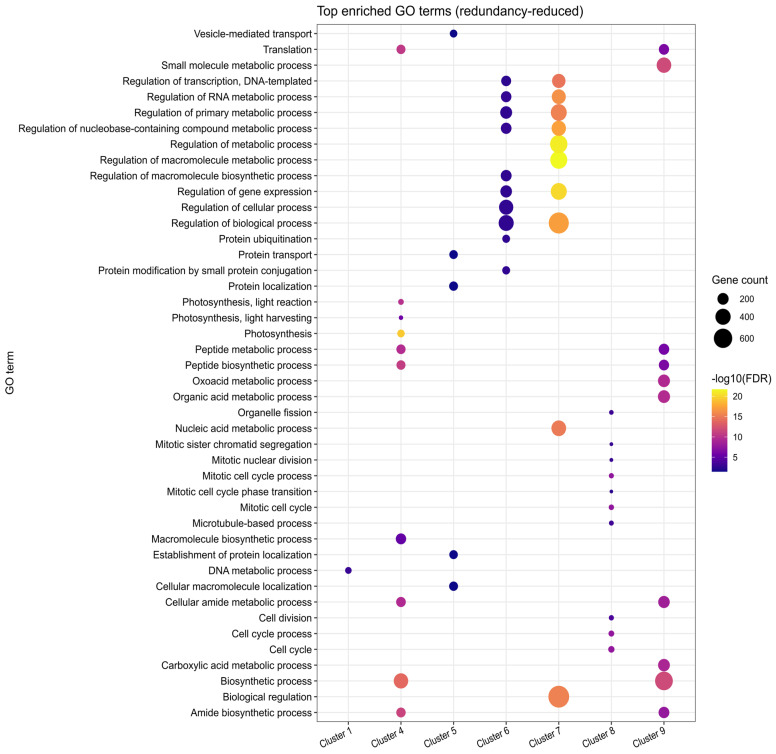
Functional enrichment bubble plot showing the top 10 enriched categories across gene expression clusters. The x-axis represents clusters, while the y-axis lists enriched functional categories (GO functional classification). Each bubble represents a specific category enriched within a given cluster. Larger bubbles indicate stronger enrichment (higher enrichment factor), while colors correspond to the statistical significance (−log10(FDR-adjusted *p*-value)), with lighter colors representing higher significance levels. Enrichment reflects organ-level trends derived from whole-fruit RNA-seq data, without implying cell-type-specific expression. Complete enrichment analysis results are available in Supplementary File S1.

To better understand the molecular processes and key factors involved in early fruit development, we performed k-means clustering analysis for the three developmental stages (Figure 2, Supplementary File S2). Since samples comprise whole fruits, expression changes across stages reflect system-level transitions. Therefore, we avoid attributing changes to specific cell types and acknowledge that shifts in tissue proportions may contribute to the observed patterns. The clustering analysis identified nine distinct expression patterns (clusters), which highlight the dynamic and coordinated regulation of genes as the fruit transitions from cell proliferation to cell expansion. Additionally, GO enrichment analysis provided further insight into the biological processes underlying these expression patterns, which represent diverse trends of gene upregulation, downregulation, or stage-specific peaks in the 3–8 DPA interval (Figure 3, Supplementary File S2).

Cluster 1, consisting of 955 genes, shows high expression levels at 3 and 5 DPA, followed by a notable decrease at 8 DPA. GO enrichment analysis revealed that a substantial portion of these genes, including at least 55 proteins involved in DNA replication, play a crucial role in cell division. Examples include DNA topoisomerase-encoding gene *SLM2ch01g00868* (*TOP6B*), a DNA gyrase subunit *SLM2ch01g03804* (*GYRA*), replication licencing genes *CDT1* (*SLM2ch06g21110*), *MCM2* (*SLM2ch11g41496*), *MCM6* (*SLM2ch02g08173*), and *MCM7* (*SLM2ch01g02921*), and DNA polymerase delta subunit *SlPOLD1* (*SLM2ch10g38688*).

Similarly, clusters 6 and 7, containing 1252 and 2038 genes, respectively, also exhibit a declining expression trend towards 8 DPA, although the decay is more gradual compared to the sharp drop observed in cluster 1. These clusters are enriched in genes involved in the regulation of RNA metabolic processes, including 144 genes in cluster 6, and 281 genes in cluster 7 associated with category GO:0016070: RNA metabolic process (Supplementary File S2).

**Figure 4:**
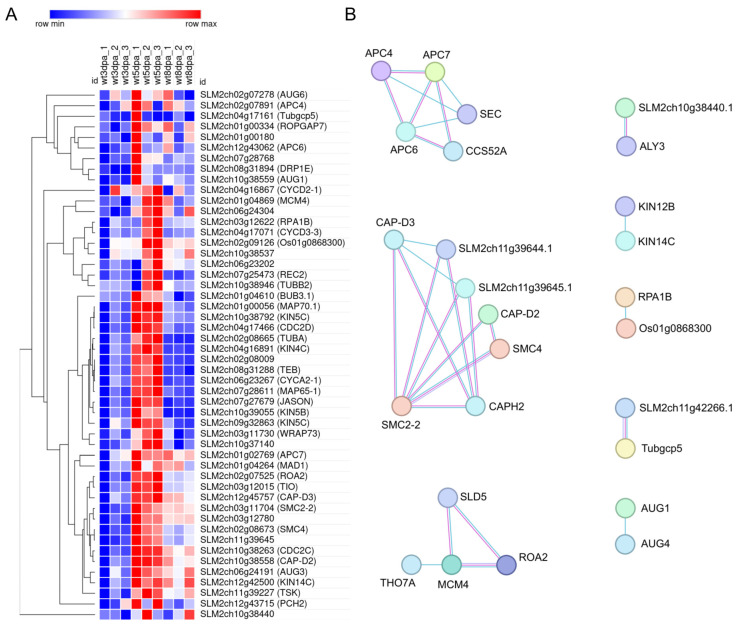
Heatmap and predicted interaction network of cell cycle–related genes from cluster 8. (**A**) Heatmap showing the expression profiles of 50 cell cycle related genes from cluster 8 across the three developmental stages. Gene symbols are provided in parentheses for annotated genes. The heatmap was generated using the Morpheus tool (https://software.broadinstitute.org/morpheus, accessed on 10 November 2025). (**B**) Predicted protein–protein interaction network based on conserved interactions from homologs in other organisms, as retrieved from the STRING database. Line thickness represents the strength of evidence supporting each interaction.

A distinct set of genes shows a peak at 5 DPA (cluster 8, 605 genes), with the cell cycle being the top-enriched category, including 50 genes (Figure 3 and Figure 4A). A predicted protein-protein interaction network of genes in this cluster, based on conserved interactions from the STRING database (Figure 4B) highlights their potential roles in DNA replication, particularly during the S phase and mitosis, including members of the Anaphase Promoting Complex/Cyclosome (APC/C). Genes in this cluster that can be directly associated with the S phase are replication licensing factors *MCM3* (*SLM2ch02g07525*) and *MCM4* (*SLM2ch01g04869*)*,* and DNA polymerase alpha genes *POLA1* (*SLM2ch02g09126*) and *POLA2* (*SLM2ch05g18343*); mitosis-related genes include *SlCDKB;1* (*SLM2ch10g38263*), *SlCDKB;2* (*SLM2ch04g17466*), *SlCycA2* (*SLM2ch06g23267*), *SlCycB3;1* (*SLM2ch07g28768*), *MAD1* (*SLM2ch01g04264*), *BUB3* (*SLM2ch01g04610*), *RAD9A* (*Solyc10g044780*), with APC/C components *APC6* (*SLM2ch12g43062*) and *APC7* (*SLM2ch01g02769*).

Clusters 4, 5, and 9 represent the largest expression groups, all exhibiting an upward trend in gene expression toward the 8 DPA timepoint (Figure 2). Cluster 5 (1349 genes) displays a more pronounced increase between 5 and 8 DPA and is enriched for protein transport functions, with 105 genes associated with this GO term (Figure 3). Representative genes in this cluster include components of the Sec machinery, such as *SEC23* (*SLM2ch01g03047*); *SEC24-like* (*SLM2ch02g08176*); *SEC31-like* (*SLM2ch01g03268*), as well as transport-related proteins including *Importin-α* (*SLM2ch01g02546*), *Importin-β1* (*SLM2ch06g22754*); *Transportin-3* (*SLM2ch06g23582*), and exportin *XPO1/HASTY* (*SLM2ch01g03918*). In contrast, cluster 4 (1344 genes) exhibits a more gradual expression transition and is enriched for photosynthesis-related processes (76 genes) as well as broader cellular metabolic functions (667 genes) (Figure 3). Notable photosynthesis-associated genes in this cluster include 10 components of photosystem I (PSI), six of Photosystem II (PSII), 15 chlorophyll a/b-binding proteins, and seven enzymes of the Calvin–Benson–Bassham (CBB) cycle.

Cluster 9, the largest cluster comprising 2332 genes, is enriched for multiple metabolic processes (Figure 3; Supplementary File S2), including energy metabolism. This includes genes involved in mitochondrial oxidative phosphorylation, such as components of Complex I (*SLM2ch01g04824*, *SLM2ch10g37930*), Complex II (*SLM2ch02g08444*), Complex III (Rieske protein, *SLM2ch11g41553*), Complex IV (*SLM2ch02g09005*), and ATP *synthase* (*SLM2ch04g13618*, *SLM2ch05g17842*), as well as electron carriers like cytochrome c (*SLM2ch01g04279*). Core steps of glycolysis and tricarboxylic acid (TCA) are also represented, including *Phosphofructokinase* (*SLM2ch11g39552*), *Pyruvate kinase* (*SLM2ch03g09589*), *Glyceraldehyde-3-Phosphate Dehydrogenase* (*SLM2ch03g12260*), *Pyruvate Dehydrogenase subunit E1α* (*SLM2ch04g13473*), *citrate synthase* (*SLM2ch01g02461*), and *Malate dehydrogenase* (*SLM2ch11g39422*).

### 2.4. Different Transcription Factors May Regulate Early Fruit Development

**Figure 5:**
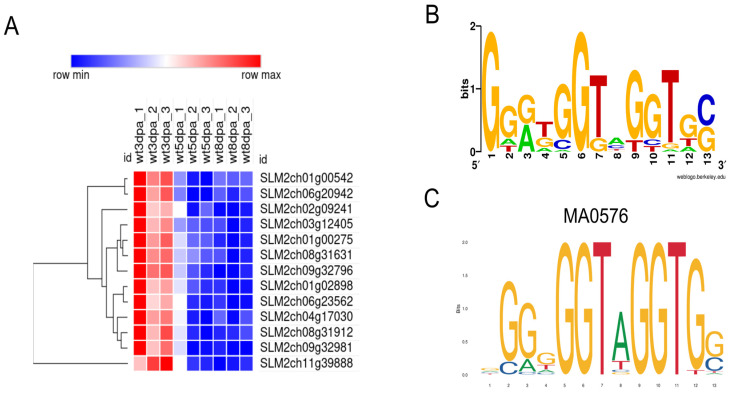
Predicted targets of the MYB TF SLM2ch04g16973 in Cluster 6. (**A**) Heatmap of target gene expression at all three timepoints; (**B**) Sequence logo of the motifs found in the 2 kb upstream sequence of the target genes; (**C**) Predicted binding motif for TF SLM2ch04g16973, from the JASPAR annotation of gene *Solyc04g077260*. Motifs were identified using FIMO5.5.7 against JASPAR and CIS-BP database for *S. lycopersicum* TFs. The heatmap was produced with Morpheus (https://software.broadinstitute.org/morpheus/, accessed on 10 November 2025).

To investigate the transcription factors (TFs) potentially regulating early fruit development, we analyzed the putative promoter regions (2 kb upstream of coding sequences) of co-expressed genes within each cluster for enrichment of TF binding motifs (Supplementary File S3). Interestingly, only one TF with significantly enriched motifs (Bonferroni-Hochberg adjusted *p*-value < 0.05) was the MYB family member SLM2ch04g16973, whose binding motifs were found in the upstream regions of 13 genes in Cluster 6 (Figure 5 and Table 2). This TF corresponds to the gene *Solyc04g077260* in the ITAG4.0 annotation of the Heinz tomato genome, and its function remains uncharacterized. The predicted targets of SLM2ch04g16973 include genes with diverse functions, such as protein kinases, RNA-binding proteins, proteasome-related proteins, and a histone acetyltransferase (Table 2). This TF was filtered out in the DE analysis due to its higher adjusted *p*-value (0.023) in the 3 vs. 5 DPA comparison, but it is slightly more expressed at 3 DPA than at both 5 and 8 DPA (Supplementary Figure S6).

To gain further insights into the transcriptional regulatory landscape during early fruit development in Micro-Tom tomato, we focused on the expression patterns of TFs across developmental stages. Of the 1623 predicted TFs in the Micro-Tom genome, 586 (36.1%) were detected in our filtered dataset, and these were distributed among the nine expression clusters (Supplementary Figure S7, Supplementary File S3). Among the detected TFs, several families such as bHLH, MYB, NAC, WRKY, ERF, C2H2, and HD-ZIP were represented by many members (Supplementary File S3), reflecting their broad regulatory functions. For instance, the Auxin Response Factor (ARF) and C3H zinc finger families showed strong representation in Cluster 1, which is characterized by higher expression at 3 DPA (Supplementary Figure S7). Although many TF families were broadly represented across clusters, a hypergeometric enrichment analysis revealed that only members of the MIKC-MADS family were significantly overrepresented in a specific cluster, Cluster 9, which comprises genes with increasing expression over time (Supplementary Figure S7). Of the 15 MIKC-MADS genes expressed in our dataset, seven exhibit the low-mid-high expression profile characteristic of cluster 9. These include *MADS-box Protein 6* (*TM6-SLM2ch02g08376)*, *MADS-box protein TM29* (*SLM2ch02g08779*), *FRUIFULL-like 2* (*FUL2-SLM2ch03g12599*), *MADS-box protein EJ2* (*SLM2ch03g12600*), *MADS-box protein 5* (*TM5*-*SLM2ch05g18516*), *TOMATO AGAMOUS-LIKE 1 (TAGL1; SLM2ch07g28242*), *MADS-box protein 8* (*MBP8-SLM2ch12g45331*), and *AGAMOUS-like 66* (*AGL66; SLM2ch07g27975*).

### 2.5. Integration of Metabolite Clusters with Gene Expression

**Figure 6:**
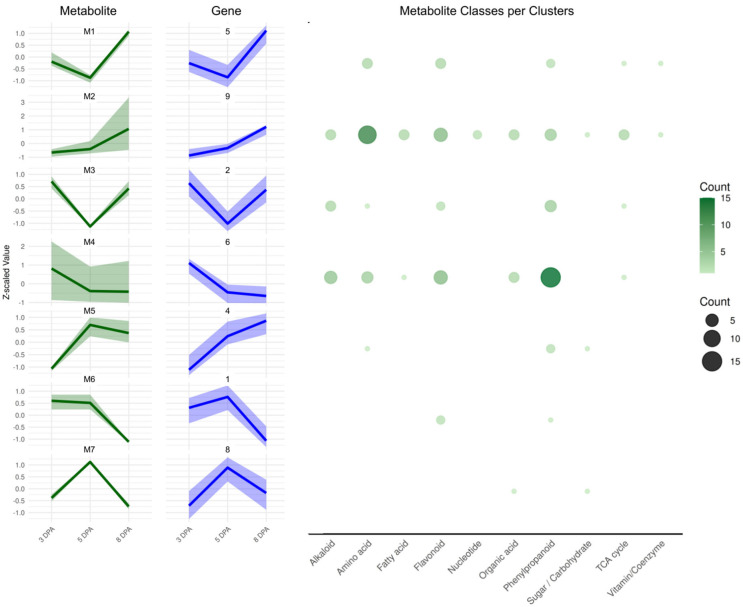
Clustering analysis of metabolites during three developmental stages of tomato fruits (3 DPA, 5 DPA, and 8 DPA). Clusters are labeled from Cluster M1 through Cluster M7 (left, green). Correlated gene clusters are pictured on next to each metabolite cluster, in blue. Gene and metabolite cluster associations were determined using Spearman correlation between average Z-score profiles. The y-axis indicates normalized expression changes, while the x-axis marks the time points. In the right, the number of metabolites in each cluster are plotted in representative functional classes. The complete results are in Supplementary File S4.

To better understand the coordination between metabolic and transcriptional programs during early fruit development, we integrated our gene expression clusters with the metabolite profiles reported by [29]. In that study, 72 metabolites identified via LC-MS and 46 via GC-MS were quantified at 3, 5, and 8 DPA in the same biological system. By leveraging this dataset, we aimed to identify temporal relationships between metabolite accumulation and gene expression, which could point to co-regulated pathways or feedback mechanisms linking metabolism to developmental gene regulation. Using hierarchical clustering, we organized these metabolites into seven distinct clusters based on their temporal patterns (Figure 6, Supplementary File S4). These metabolite profiles closely mirrored gene expression dynamics during tomato fruit development. For all clusters discussed below, metabolite-transcript concordance is reported at the organ level, as RNA-seq bulk data do not provide spatial resolution.

Cluster M1 includes metabolites such as serine, asparagine, several flavonoid derivatives, tryptophan, and succinic acid. These compounds correspond to gene cluster 5, which follows a “mid/low–low–high” expression pattern, peaking at 8 DPA. GO enrichment analysis of this gene cluster highlights functions related to protein localization and vesicle-mediated transport (Figure 3), with representative components including importins, elements of Signal Recognition Particle (SRP), vesicle coat and trafficking proteins such as COPII subunits (SEC23/SEC24/SAR1), COPI subunits (α/ζ), clathrin adaptors, Rab GTPases, SNARE proteins (Soluble NSF Attachment protein Receptors), and Sec1/Munc18 (SM) family members.

Cluster M2 comprises primary metabolites, such as quinic acid, GABA, several amino acids, and energy-related compounds including pyruvate, 2-oxoglutarate, fumarate, and glyoxylate. These metabolites correspond to gene cluster 9, which exhibits a “low–mid/low–high” expression pattern and is enriched for biosynthetic and energy metabolism processes, particularly translation and small molecule synthesis. Several genes within this cluster map directly to the measured metabolites.

For GABA metabolism, these include two GABA decarboxylase encoding-genes (*SLM2ch01g00027* and *SLM2ch03g12146*), a *GABA permease* (*SLM2ch05g17865*), and *Aminoaldehyde Dehydrogenase 2* (*SLM2ch03g12504*). For central carbon metabolism, enzymes include *Pyruvate kinase (PK, SLM2ch01g04585),* subunits of the Pyruvate dehydrogenase complex, *E1α (SLM2ch04g13473), E1β (SLM2ch04g13714), E2 (SLM2ch05g17943), and E3 (SLM2ch01g04121),* as well as *Isocitrate dehydrogenase (IDH, SLM2ch02g08555), 2-Oxoglutarate dehydrogenase (OGDH, SLM2ch04g13893), Succinate dehydrogenase (SDH, SLM2ch02g08444), Malate dehydrogenase (MDH, SLM2ch02g07060* and *SLM2ch03g12699),* and *Phosphoenolpyruvate carboxylase (PEPC, SLM2ch04g13565)*. Cluster M3 includes hydroxybenzoic acid-hexose, caffeoyl-hexoside 5, malic acid, and several glycosylated compounds such as feruloyl-glucoside 3. These correspond to gene cluster 2, which exhibits a “high–low–high” expression pattern. GO enrichment analysis indicates that cluster 2 is associated with phenylpropanoid biosynthesis and redox-related metabolic processes, consistent with the accumulation of phenolic glycosides. Representative genes involved in phenylpropanoid metabolism include *Phenylalanine Ammonia-Lyase (PAL; SLM2ch03g09958), 4-Coumarate:CoA ligase (4CL; SLM2ch06g22199* and *SLM2ch02g08238), Hydroxycinnamoyl-CoA Shikimate/Quinate Hydroxycinnamoyltransferase (HCT, SLM2ch03g12839), Cytochrome P450 Monooxygenase (P450/CYP, SLM2ch03g12278* and *SLM2ch06g24027), NADPH-Cytochrome P450 Reductase (CPR, SLM2ch07g26113), UDP-Glycosyltransferase (UGT; SLM2ch02g07465* and *SLM2ch04g16942),* and *Glutathione S-Transferase (GST; SLM2ch07g28292* and *SLM2ch08g32223)*. These genes are likely involved in the biosynthesis and glycosylation of hydroxycinnamic acid derivatives observed in this cluster. In addition, genes associated with redox regulation and organic acid metabolism were identified, including *Phosphoenolpyruvate Carboxylase (PEPC; SLM2ch12g43054*) and *PEPC kinase* (*PPCK; SLM2ch04g13826*) together with NAD(P)H-generating redox enzymes such as *6-Phosphogluconate Dehydrogenase (6-PGDH; SLM2ch12g44745)*, *Glycerol-3Phosphate Dehydrogenase (GPDH; SLM2ch01g03278)*, *Lactate Dehydrogenase (LDH-SLM2ch08g32018).* Together, these genes support active turnover of malic acid and other organic acids during initial stages of fruit development.

Metabolite cluster M4 is composed of caffeoyl derivatives, quinic acid isomers, flavonoids, and coumaric acid derivatives. These metabolites are associated with gene cluster 6, which displays a “high–low–low” expression pattern, characterized by strong early expression that declines during later developmental stages. While genes in cluster 6 are predominantly enriched for functions related to protein modification (including ubiquitination and SUMOylation) and transcriptional regulation, several genes involved in phenylpropanoid and shikimate pathway biosynthesis are also present. These include *PAL* (*SLM2ch05g20828*), *4CL* (*SLM2ch03g12270, SLM2ch03g12861*), *Cinnamate 4-hydroxylase* (*C4H; SLM2ch06g24142*), *Caffeoyl-CoA O-methyltransferase* (*CCoAOMT; SLM2ch10g37776*), *Flavonoid 3’-monooxygenase-like* (*F3’H; SLM2ch11g40926*), glycosyltransferases (*SLM2ch09g35841*, *SLM2ch11g39697*, and *SLM2ch10g38445*), *Dehydroquinate synthase* (*DHQS; SLM2ch02g08300*), and *Shikimate Kinase* (*SK; SLM2ch04g15686*)

Cluster M5 includes metabolites such as beta-alanine, caffeoyl-glucarate, fructose, and feruloyl-glucoside 1. These metabolites align with gene cluster 4, which follows a “low–mid–high” expression pattern. Genes within this cluster potentially linked to the observed metabolites include glycosyltransferases (*SLM2ch01g03657*, *SLM2ch07g27938*, *SLM2ch07g27627*, *SLM2ch07g27629*, and *SLM2ch10g36500*), invertases (*SLM2ch01g04926*, *SLM2ch06g23223*), and sugar transporters (*SLM2ch04g17254*, *SLM2ch07g25553*).

Metabolite cluster M6 includes Que-hexose, quinic acid 3-caffeoyl trans, and quercitrin, which are associated with gene cluster 1. This cluster displays a “high–high–low” expression pattern, with early gene expression linked to DNA metabolism and cell proliferation. Notably, cluster 1, also contain genes involved in phenylpropanoid and flavonol-biosynthesis, including *PAL* (*SLM2ch10g39042*), *4CL* (*SLM2ch01g02500*), *2-oxoglutarate-dependent dioxygenase* (*2-OGDD; SLM2ch01g02212*), and a *Shikimate dehydrogenase* (*SDH; SLM2ch06g24311*). In addition, the presence of *hexosyltransferases* (*SLM2ch01g05027, SLM2ch11g39854*) and *UDP-glucose pyrophosphorylase* (*UGPase-SLM2ch06g22670*), suggests plausible biosynthetic routes leading to the metabolites observed in cluster M6.

Finally, cluster M7 includes salicylic acid (SA) and sucrose, and corresponds to gene cluster 8, which display a “low–high–low” expression profile, with a transient peak at 5 DPA. As described above, GO enrichment analysis of this gene cluster highlights functions related to cell cycle regulation (Figure 4). However, other genes in cluster 8 can also be associated with SA-related pathways, including *PAL1* (*SLM2ch03g09966*) and *WRKY70* (*SLM2ch03g11880*). Genes related to sucrose metabolism are also present, including *sucrose synthase* (*SLM2ch02g08092*), *invertases* (*SLM2ch11g42032, SLM2ch09g32865*), *vacuolar glucose transporter* (*SLM2ch03g11354*), and *galactinol–sucrose galactosyltransferase* (*SLM2ch07g25633*).

## 3. Discussion

In this study, our organ-level transcriptomic analysis of Micro-Tom tomato revealed a dynamic and tightly regulated gene expression program during early fruit development. Our whole-fruit profiles recapitulate established early fruit developmental trajectories: prominent mitotic signatures at the earlier stages, followed by increasing expression of genes associated with cell expansion, endoreduplication, and carbohydrate metabolism between 5–8 DPA, in agreement with tissue-resolved transcriptomic datasets and classical histological observations [14,30,31,32]. This pattern aligns with canonical models of tomato fruit metabolism, which emphasize early carbon reprogramming and a developmental shift from partial photosynthetic capacity to increasingly heterotrophic growth [33,34]. Similar trends in early carbon metabolism were also observed in the *Chico* cultivar, where starch and sucrose metabolism was the most dynamically regulated pathway from 0–12 DPA at organ level (bulk RNA-seq), with several sugar- and cell-wall-related enzymes [35], supporting our observed shift during the 5–8 DPA window. Histological analysis of tomato line Wva106 further showed that cell division and expansion occur in parallel until 9 DPA [36].

We identified nine distinct gene expression clusters that reflect the developmental progression of early fruit growth. Clusters expressed during the earliest stages were enriched for DNA and RNA metabolic processes and replication licensing, consistent with active cell proliferation. In contrast, clusters peaking later were enriched for genes involved in biosynthesis process, energy metabolism, and cell wall modification, marking the transition toward cellular expansion. Therefore, we used these clusters as a background to compare developmental timing across cultivars and to contextualize metabolite accumulation within the critical 3–8 DPA window, where regulatory shifts in both transcriptional programs and metabolic pathways converge.

Comparisons with other tomato cultivars and related species highlights both conserved regulatory pathways and Micro-Tom-specific features. Our findings corroborate with previous studies showing that hormone signaling genes, particularly auxin and gibberellin components, as well as core cell-cycle regulators, play central roles in orchestrating the early developmental transition [14,31,34,36]. Clusters with high expression at 3 and 5 DPA are enriched for DNA and RNA metabolism, as well as replication licensing factors, consistent with the intense mitotic activity that begins immediately after fertilization [14,31]. In agreement with histological data, the most pronounced transcriptional shift occurs between 5 and 8 DPA (Figure 1), marking the transition from cell division-driven growth to cell expansion. This shift is accompanied by a decline in mitotic gene expression and the upregulation of pathways associated with cellular enlargement, carbohydrate metabolism, and structural modification [26,30]. In Micro-Tom, the pericarp cell layers increase by 4–5 DPA and cell expansion becomes apparent by 7 to 8 DPA, while cell division persists in the outer mesocarp layers [27]. This developmental progression aligns closely with the molecular shift observed between 5–8 DPA in our transcriptomic data, however this transition time can vary across genetic backgrounds [8,30], reinforcing that the trends captured by bulk RNA-seq reflect an organ-level developmental transition whose inflection can be cultivar-dependent. At the tissue level, the same fertilization signals are proposed to synchronously trigger mitosis, endoreduplication, and early expansion in specific pericarp layers [36], providing a mechanistic backdrop for our observations. Importantly, our experimental design closely parallels the stage groupings seen in the ‘Chico’ cultivar study, which showed similar transcriptomic clustering across 3–5 DPA, 7–9 DPA, and 12 DPA, reinforcing that the early division-expansion molecular shift is a common feature across different genetic backgrounds [35].

In this context, hormone signaling modules provide a mechanistic framework for the observed decline in mitotic gene expression and the rise of pathways associated with cell expansion. Zhang et al. [14] reported spatially distinct expression pattern of auxin biosynthesis and transport and ARF/IAA transcriptional regulators in the ‘Moneymaker’ [11]. The auxin-gibberellin crosstalk mediated by DELLA-ARF/IAA proteins is essential to fruit initiation, which happens at 0–1 DPA [11,37,38,39,40]. For instance, *SlARF7* and *SlARF9* have been shown to modulate fruit set and early pericarp cell division, respectively, after fruit initiation [38,39]. In tissue-resolved datasets, *SlARF7* peaks in the ovule at 0 DPA, followed a sharp decline post-fertilization, whereas *SlARF9* shows increasing expression throughout development in both the ovule and the ovary wall/pericarp [14]. Our whole-fruit Micro-Tom transcriptome profiles, *SlARF7* (SLM2ch07g27516; Cluster 7) shows a similar trend, with highest expression at 3 DPA followed by a decline at 8 DPA), while *SlARF9* (SLM2ch08g32375; Cluster 1) peaks at 5 DPA. Thus, the overall trends are broadly consistent, with minor differences in timing or expression magnitude likely attributable to whole-fruit averaging and potential cultivar-specific effects.

Consistent with the observed hormonal cues, the transcription-factor (TF) landscape reveals shared regulatory modules and line-specific tuning. Comparative analysis of TF expression supports a pattern of overlap and divergence. Both, Zhang et al. [14] and our study identified members of MADS-box and AP2 families as key regulators during early development. In our dataset, MIKC-MADS TFs showed later upregulation, with peak expression at 8 DPA, consistent with the findings of [14], who reported highest expression of M-type MADS expression in ovules at 5 DPA, the latest time point sampled in their study. In contrast, TF families such as TALE, which were prominent in their pericarp-specific analysis, were underrepresented in our whole-fruit dataset, likely reflecting differences in tissue resolution and/or genetic background. Nonetheless, these findings suggest a conserved core regulatory network modulated by cultivar-specific transcriptional programs. To investigate whether these transcriptional dynamics are mirrored at the metabolic level, we integrated metabolite profiles measured at corresponding developmental stages.

Integration of transcriptomic and metabolomic data yielded several key insights. In our profiles, Cluster 8 displayed a peak at 5 DPA, coinciding with elevated levels sucrose and salicylic acid (Figure 2 and Figure 6). Sucrose is well-known to act as a metabolic checkpoint that promote the G1/S transition during the cell cycle and, in Arabidopsis, has been shown to rapidly induce D-type cyclins in sugar-starved cells [41]. Additionally, developing fruits import sucrose and convert it to starch, a process supported by the cell-type-specific expression of carbon metabolism genes across different pericarp tissues [32]. Early gene-metabolite correlation networks in expanding tomato tissues have also identified regulatory hubs linking sugars/organic acids with TFs across mesocarp and locular tissues [42], further supporting the concordance observed between our transcript and metabolite profiles. Moreover, the upregulation of cell wall and carbon-related genes at 8 DPA agrees with prior evidence in the ‘Chico’ cultivar, which demonstrate that starch, sucrose, and wall modifying enzymes (invertases, pectin methylesterases) are involved during early tomato fruit development [35]. These findings support a model in which sucrose availability during this stage functions not only an energy source, but also as a developmental cue for cell proliferation in tomato fruit.

Salicylic acid may also play a regulatory role at this stage. While high SA levels are associated with stress responses and growth inhibition, moderate concentrations can promote cell division, particularly through crosstalk with auxin and ROS signaling [43]. Given its peak at 5 DPA, SA may act alongside sugars to fine-tune the balance between proliferation and differentiation. As mitotic activity decline and wall/carbon pathways increase, the network shifts toward energy supply and biomass accumulation, with organic acids (citrate, malate, fumarate) enriched in locular tissue and hexoses and starches more abundant in mesocarp during expansion [44].

Cluster 9 peaks at 8 DPA and contains primary metabolites (quinic acid, GABA, amino acids) and energy-related compounds (pyruvate, 2-oxoglutarate, fumarate, and glyoxylate). Most amino acids accumulated at 8 DPA, aligning with the onset of cellular expansion and increased metabolic demand. Notably, flavor-associated amino acids such as glutamate and aspartate, though abundant in later ripening stages [45], also showed early fluctuations, suggesting that amino acid metabolism is already active during the initial phases of fruit development, well before its sensory relevance emerges.

Metabolite cluster M2, which includes key carbon intermediates like 2-oxoglutarate, fumarate, and pyruvate, correlated with gene Cluster 9, which is enriched in genes linked to carbon metabolism. This association points to increased mitochondrial activity and metabolic flux, supporting both biosynthesis and energy production during cell-wall development and storage-compound accumulation [46]. Together, these transcript-metabolite associations reflect a coordinated transition from hormone-primed proliferation to carbon-supported expansion.

Future studies employing spatial transcriptomics and metabolomics could resolve tissue-specific gene-metabolite interactions that are masked in whole-organ profiling. Such approaches may clarify whether observed differences in hormone signaling and sugar/amino acid metabolism reflect adaptive responses, tissue dilution effects, or inherent to the cultivar. In Micro-Tom, our organ-level profiles at 3, 5, and 8 DPA recapitulate key early fruit transitions and reveal stage-matched metabolite concordance, providing a cultivar-specific, temporal reference framework. Spatially resolved data can build upon this foundation to uncover localized regulatory networks. A deeper understanding of these regulatory mechanisms could inform targeted breeding strategies to improve fruit quality, size, and stress resilience in tomato.

## 4. Materials and Methods

### 4.1. Plant Material and Experimental Design

Tomato (*S. lycopersicum* cv. Micro-Tom) plants were grown under controlled conditions of 24/18 °C day/night, 16 h photoperiod, and relative humidity of 50–60% in a greenhouse. Plants were irrigated daily. Plants were tagged at anthesis (0 DPA), and sampling occurred at 3, 5, and 8 DPA. For each timepoint, three pools of 12 whole fruits were collected and immediately frozen in liquid nitrogen, ground in pestle and mortar, and stored at −80 °C. Sampling was performed in the similar hours of the day to minimize diel effects. The 3–8 DPA window was chosen to span the documented division-dominated to expansion-dominated growth in early tomato fruit [26]. Our design targets organ-level trajectories across this interval.

### 4.2. RNA Sequencing

Sequencing libraries were obtained from our previous work [29]. Briefly, stranded total RNA libraries were prepared fyourrom samples of developing Micro-Tom fruits collected at 3, 5, and 8 DPA, using three biological replicates per stage, each consisting of a pool of 12 fruits. Sequencing was performed by Fasteris Co., Ltd. (Plan-les-Ouates, Switzerland) using the Illumina NovaSeq 6000 platform, generating 150 base pairs (bp) paired-end reads. Raw reads were processed with TrimGalore (version 0.6.10) (github.com/FelixKrueger/TrimGalore), using default parameters for adapter trimming and quality filtering. Quality assessment of the processed reads was conducted using FastQC (version 0.12.1) (bioinformatics.babraham.ac.uk/projects/fastqc) and RSeQC (version 5.0.1) [47] before proceeding to downstream analyses.

### 4.3. Read Mapping and Transcript Quantification

Processed reads were mapped to the most recent assembly of the Micro-Tom genome [19] (NCBI accession GCF_036512215.1) using the STAR aligner (version 2.7.11b) [48], with default parameters optimized for paired-end reads. Gene quantification was achieved by enabling the “–quantMode GeneCounts” option during mapping, which generates read counts per gene directly from the aligner. The count table was then filtered to eliminate low-expressed genes by eliminating genes with less than 10 total reads across at least 3 samples.

### 4.4. Differential Expression Analysis

Differential expression analysis was conducted using the RNFuzzyApp package (https://gitlab.com/habermann_lab/rna-seq-analysis-app, accessed on 5 June 2024) in R (version 4.3.3). Raw gene counts were normalized using the Trimmed Mean of M-values (TMM) method [49]. The resulting expression matrix was analyzed using the TCC method [50], with statistical testing performed by DESeq2 (version 1.48.2) [51]. A False Discovery Rate (FDR) cutoff of 0.01 was applied to identify significantly differentially expressed genes, with |log2FC| > 1.5.

### 4.5. Expression Clustering

Clustering of expression patterns was performed using a separately filtered set optimized for clustering. Starting from the TMM-normalized counts, genes were retained if they had a minimum of 5 read counts in every sample, resulting in 11,035 consistently expressed genes. The expression values were transformed using a log2(x + 1) function to stabilize variance and then standardized using Z-score normalization. The average expression value for each gene at each time point was calculated for clustering purposes. K-means clustering was executed in R using the kmeans function, specifying nine clusters (k = 9) and setting the maximum number of iterations to 50. A random seed of 123 was used to ensure reproducibility of the clustering results. The k = 9 value was selected based on elbow criterion and cluster stability across 100 random starts (testing k = 2–15).

### 4.6. Functional Enrichment Analysis

Functional enrichment analysis was conducted using the STRING database (version 12.0) [52] and the clusterProfiler (version 4.16.0) R package [53]. The proteome encoded by the Micro-Tom genome was annotated within STRING to facilitate the analysis and is accessible at https://version-12-0.string-db.org/organism/STRG0A81OUP(accessed on 10 November 2025). Gene IDs from each cluster were uploaded to the STRING workspace to create gene sets. For the DE genes, the enricher function of clusterProfiler was used with the STRING annotation. The total number of expressed genes from the normalized, filtered expression matrices served as the background for statistical enrichment analysis. Overrepresented Gene Ontology (GO) terms and pathways were identified for each cluster using built-in enrichment functions, applying statistical significance cutoffs of *p* < 0.05 and Benjamini–Hochberg FDR < 0.05 in all cases.

### 4.7. Transcription Factor Prediction and Analysis

Transcription factors (TFs) were identified through BLASTP (version 2.17.0+) similarity searches against known TFs in the PlantRegMap database [54] and via de novo prediction using the planttfhunter R package (version 1.6.0) [55]. Expression values of the identified TFs were transformed using a robust Z-score (subtract median and divide by median absolute deviation). Hierarchical clustering was performed using one minus Pearson correlation as the distance metric, enabling the assessment of expression pattern similarities among TFs during fruit development stages.

### 4.8. Transcription Factor Motif Mapping and Enrichment Analysis

This analysis was performed similarly to what is described by [56]. Known TF binding motifs for *S. lycopersicum* were obtained from CisBP v2.00 [57] and JASPAR 2022 [58]. These motifs were mapped to the 2-kb upstream regions of all annotated genes in the genome using FIMO v5.5.7 [59] with default parameters. We retained the top 7000 scoring motif matches, discarding lower-scoring hits. For each TF motif, hypergeometric tests were used to assess whether the number of motif instances found in genes of a given cluster (Clusters 1–9) was significantly higher than expected based on its total number of occurrences across all expressed genes. Benjamini-Hochberg correction was applied to control the false discovery rate (FDR < 0.05). Representative sequence logos showing nucleotide frequencies at each position were generated using WebLogo (http://weblogo.berkeley.edu/logo.cgi, accessed on 10 November 2025).

### 4.9. Integration of Metabolomics Data

Normalized metabolite intensities at 3, 5, and 8 DPA [28] were scaled to Z-scores. We performed k-means clustering on the Z-scored matrix, testing k = 2–10 and selecting k = 7 based on the elbow criterion and cluster stability across 100 random starts. To link metabolite clusters (1–7) to gene clusters (1–9), we computed the Spearman correlation between the centroid profiles (mean Z-score at each timepoint) of each metabolite cluster and each gene cluster. Clusters were associated by high Spearman correlation coefficients (>0.8) supported by visual pattern confirmation.

## 5. Conclusions

In summary, our integrative analysis of transcriptomic and metabolomic data in Micro-Tom tomato revealed distinct gene expression clusters associated with key developmental transitions during early fruit development. These clusters highlight a tightly regulated progression from cell division to expansion, driven by coordinated changes in hormone signaling, transcription factor activity, and metabolic pathways. Our findings also uncover both conserved regulatory mechanisms shared with other tomato cultivars and cultivar-specific differences in transcriptional and metabolic programs.

By linking metabolite accumulation to gene expression dynamics, this study provides new insights into the molecular mechanisms regulating fruit formation. The observed associations between sugars, amino acids, and cell cycle regulators suggest potential metabolic checkpoints that influence early fruit growth. These findings lay the groundwork for future functional studies, such as mutant validation (CRISPR), chromatin accessibility assays (ChIP-seq), and spatial transcriptomics, to further dissect critical regulatory networks.

These insights can directly inform breeding programs aimed at enhancing fruit size, nutritional quality, and resilience to environmental stress, as well as guide biotechnological strategies using precision breeding techniques such as CRISPR/Cas.

## Figures and Tables

**Table 1:** Summary of RNA-seq quality and alignment statistics for Micro-Tom samples at three developmental stages.

Sample	Raw Read Pairs	Clean Read Pairs	Q30% (Before)	Q30% (After)	Uniquely Mapped %	Multi-Mapping %	Unmapped %
3DPA_R1	38,108,973	33,199,719	91.49	94.61	90.86	4.69	4.44
3DPA_R2	36,774,120	30,619,852	91.42	94.76	92.44	4.21	3.34
3DPA_R3	43,253,948	33,210,737	90.26	95.03	92.41	4.26	3.33
5DPA_R1	36,012,854	28,486,863	91.16	95.10	92.21	4.56	3.22
5DPA_R2	39,869,504	32,194,417	89.80	94.76	90.62	4.49	4.90
5DPA_R3	35,938,412	28,865,298	90.46	94.79	90.37	4.49	5.14
8DPA_R1	38,193,796	32,379,259	91.89	94.91	92.76	4.46	2.78
8DPA_R2	38,321,852	27,953,423	89.58	95.05	91.90	4.69	3.41
8DPA_R3	35,581,593	28,852,087	90.92	94.67	92.78	4.59	2.63

**Table 2:** List of the putative target genes of the MYB TF SLM2ch04g16973 in Cluster 6. Corresponding ITAG4.0 annotation and description are given, as well as the predicted motif positions in the upstream regions of each gene (relative to the start codon ATG).

Gene	ITAG4.0	Description	Number of Motifs	Position (Strand)
*SLM2ch01g00275*	*Solyc01g008120*	Histone Acetyltransferase	1	−45 to −33 (+)
*SLM2ch01g00542*	*Solyc01g010660*	Receptor-like protein kinase At3g21340	3	−85 to −73 (−), −1419 to −1407 (−), −1674 to −1662 (−)
*SLM2ch01g02898*	*Solyc01g079250*	RNA helicase DEAH-box2	1	−1249 to −1237 (+)
*SLM2ch02g09241*	*Solyc02g094610*	Transportin	1	−227 to −215 (−)
*SLM2ch03g12405*	*Solyc03g112660*	Modifier of SNC1	1	−1547 to −1535 (+)
*SLM2ch04g17030*	*Solyc04g077940*	Flowering time control protein FPA	2	−783 to −771 (−), −1076 to −1064 (−)
*SLM2ch06g20942*	*Solyc06g005500*	tomato protein kinase 1b	4	−438 to −426 (+), −868 to −856 (+), −1564 to −1552 (+), −1785 to −1773 (+)
*SLM2ch06g23562*	*Solyc06g070980*	Ubiquitin-conjugating enzyme E2 2	4	−294 to −282 (−), −476 to −464 (−), −1045 to −1033 (−), −1288 to −1276 (−)
*SLM2ch08g31631*	*Solyc08g074370*	DDB1- and CUL4-associated factor homolog 1	1	−296 to −284 (+)
*SLM2ch08g31912*	*Solyc08g077560*	ATP binding/serine-threonine kinase	2	−779 to −767 (−), −1339 to −1327 (−)
*SLM2ch09g32796*	*Solyc09g009350*	Vacuolar sorting-associated protein	2	−263 to −251 (+), −748 to −736 (+)
*SLM2ch09g32981*	*Solyc09g011320*	Serine/threonine-protein kinase-like protein	4	−191 to −179 (−), −524 to −512 (−), −677 to −665 (−), −1122 to −1110 (−)
*SLM2ch11g39888*	*Solyc11g015890*	F-box family protein	1	−815 to −803 (+)

## Data Availability

The datasets generated and/or analyzed during the current study are not publicly available, as they will be integrated with additional data for a subsequent publication.

## Supplementary Materials

The following supporting information can be downloaded at: https://www.mdpi.com/article/10.3390/plants15010137/s1, Figure S1: RNA-seq read coverage of gene bodies; Figure S2: PCA analysis of RNA-seq samples before and after normalization; Figure S3: Dotplot of GO enrichment analysis for upregulated genes in the 3 vs. 5 DPA comparison; Figure S4: Dotplots of GO enrichment analysis for up- and downregulated genes in the 5 vs. 8 DPA comparison; Figure S5: Dotplots of GO enrichment analysis for up- and downregulated genes in the 3 vs. 8 DPA comparison; Figure S6: Expression plot of the MYB family member SLM2ch04g16973 at each timepoint; Figure S7: Expression profiles of transcription factors and cluster membership across the three timepoints; File S1: Complete Differential Expression analysis results and GO enrichment results; File S2: Gene expression clustering results; File S3: Transcription factor motif enrichment analysis results; File S4: Metabolite abundance clustering results.
