# Supplementary material for: Transcriptomic Analysis of Early Fruit Development in Micro-Tom Tomato Reveals Conserved and Cultivar-Specific Mechanisms
Source: Plants (Basel). 2026 Jan 3;15(1):137. doi: 10.3390/plants15010137 (PMC12787644; doi:10.3390/plants15010137)
Supplement: Supplementary file 1 [file plants-15-00137-s001.zip › Legends for the Supplementary Figures.pdf]

Legends for the Supplementary Figures

Figure S1: A - The gene body coverage curve shows that the bulk of the reads map within the 10th to 90th percentiles of the 10,000 most expressed transcripts consistently in all samples. The curve shows an absence of 5’ or 3’ bias in the read coverage. B - A heatmap of gene body coverage of the 10,000 most expressed transcripts for all aligned reads from each library. Most of the reads fall within the 10th to 90th percentiles. The plots were generated using the script “geneBodyCoverage.py” from the RseQC package v. 5.0.1 (Wang et al., 2012).

Figure S2: Principal Component Analysis before and after TMM normalization. The similar grouping of samples show normalization did not affect sample distribution, reflecting their robustness.

Figure S3: Dotplot picturing the results of GO enrichment analysis for upregulated DEGs in the 3 vs. 5 DPA comparison.

Figure S4: Dotplots picturing the results of GO enrichment analysis for upregulated (left) and downregulated (right) DEGs in the 5 vs. 8 DPA comparison.

Figure S5: Dotplots picturing the results of GO enrichment analysis of upregulated (left) and downregulated (right) DEGs in the 3 vs. 8 DPA comparison.

Figure S6: Normalized counts of gene SLM2ch04g16973 at 3, 5, and 8 DPA. Error bars represent the standard deviation from three biological replicates.

Figure S7: Expression profiles of 586 TFs at 3, 5, and 8 DPA in Micro-Tom tomato. A: Heatmap of all TFs and their association with expression patterns of all genes; B: Expression profiles of 16 MIKC_MADS TFs, with corresponding ITAG4.1 annotations and cluster memberships; C: Expression profiles of 18 genes encoding ARF TFs; D: Expression profiles of 30 genes encoding C3H TFs. Heatmaps were produced with the Morpheus tool (https://software.broadinstitute.org/morpheus/).
