# Supplementary figures and images for: Transcriptomic Analysis of Early Fruit Development in Micro-Tom Tomato Reveals Conserved and Cultivar-Specific Mechanisms
Source: Plants (Basel). 2026 Jan 3;15(1):137. doi: 10.3390/plants15010137 (PMC12787644; doi:10.3390/plants15010137)

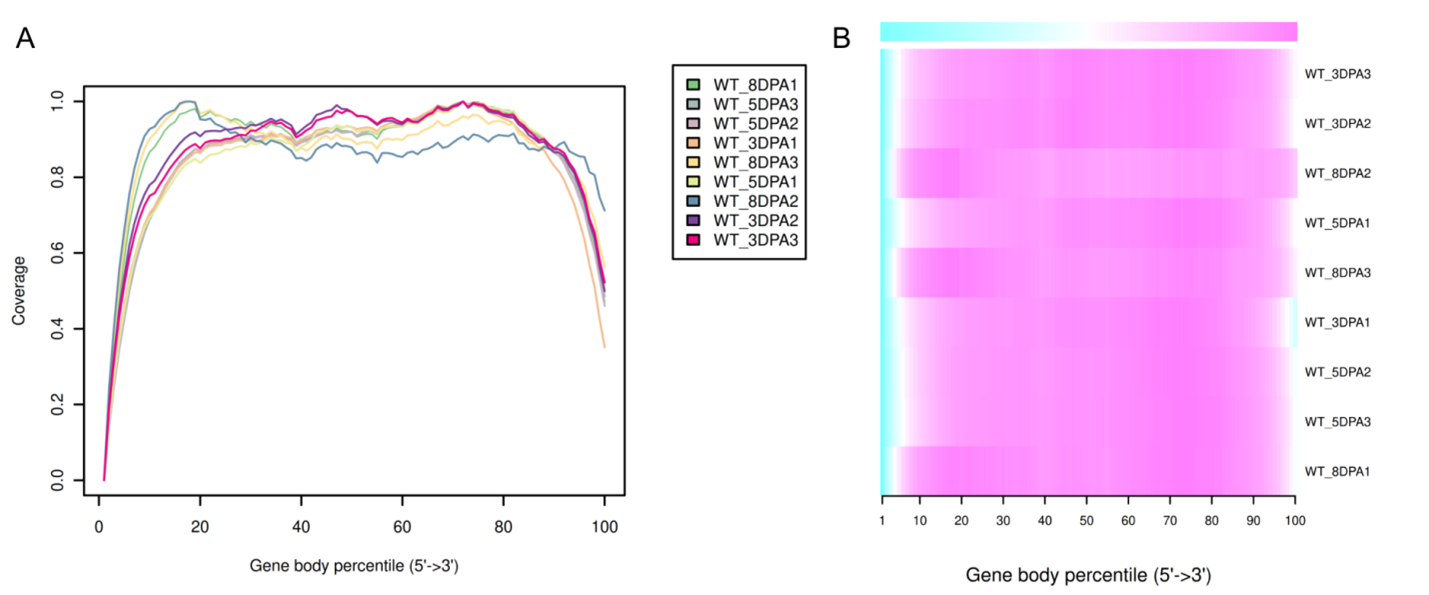

Supplement: Supplementary file 1 [file plants-15-00137-s001.zip › Figure S1.png]

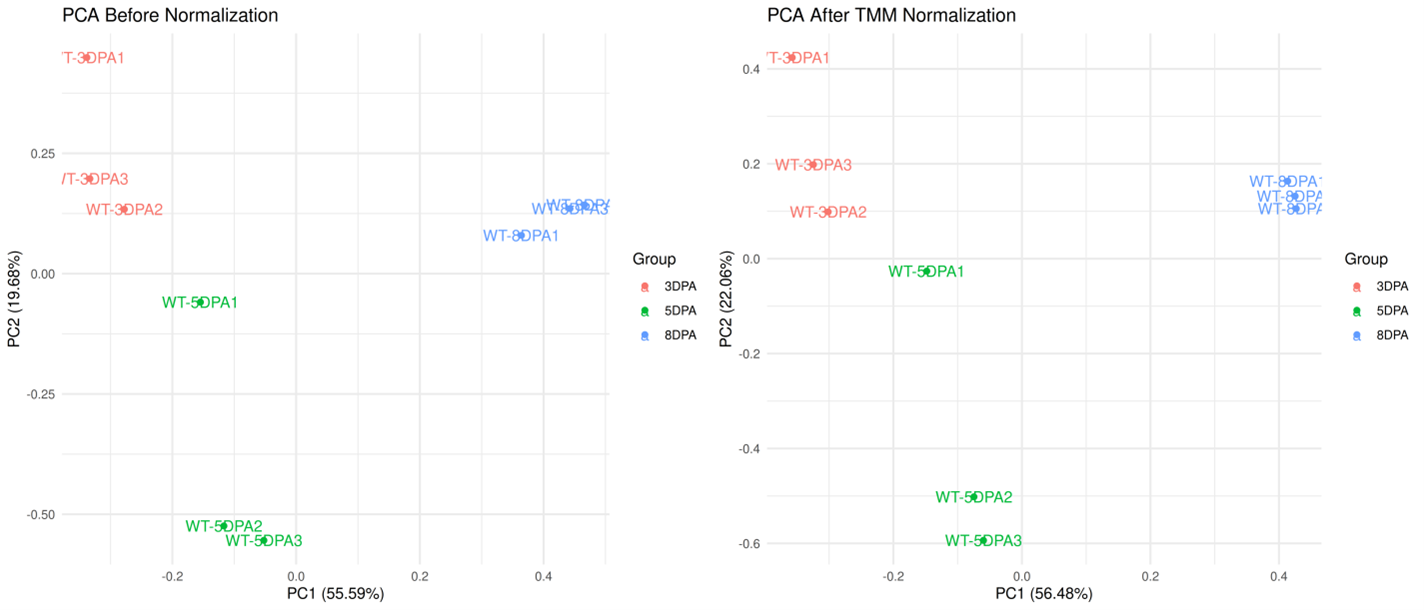

Supplement: Supplementary file 1 [file plants-15-00137-s001.zip › Figure S2.png]

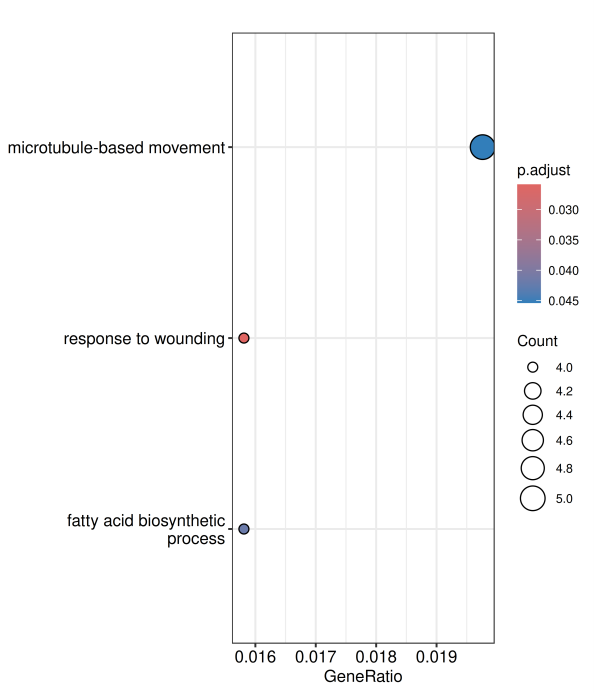

Supplement: Supplementary file 1 [file plants-15-00137-s001.zip › Figure S3.png]

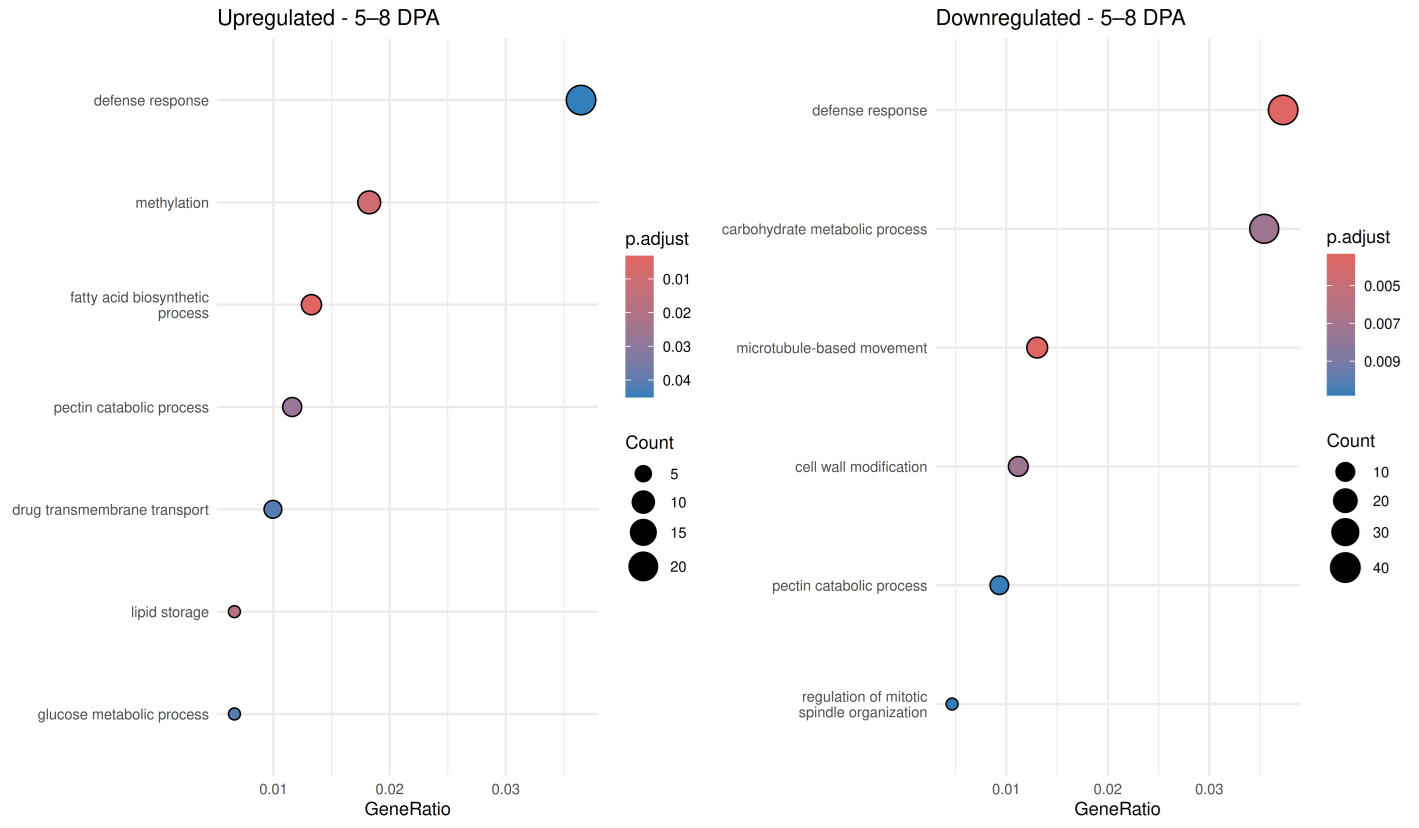

Supplement: Supplementary file 1 [file plants-15-00137-s001.zip › Figure S4.png]

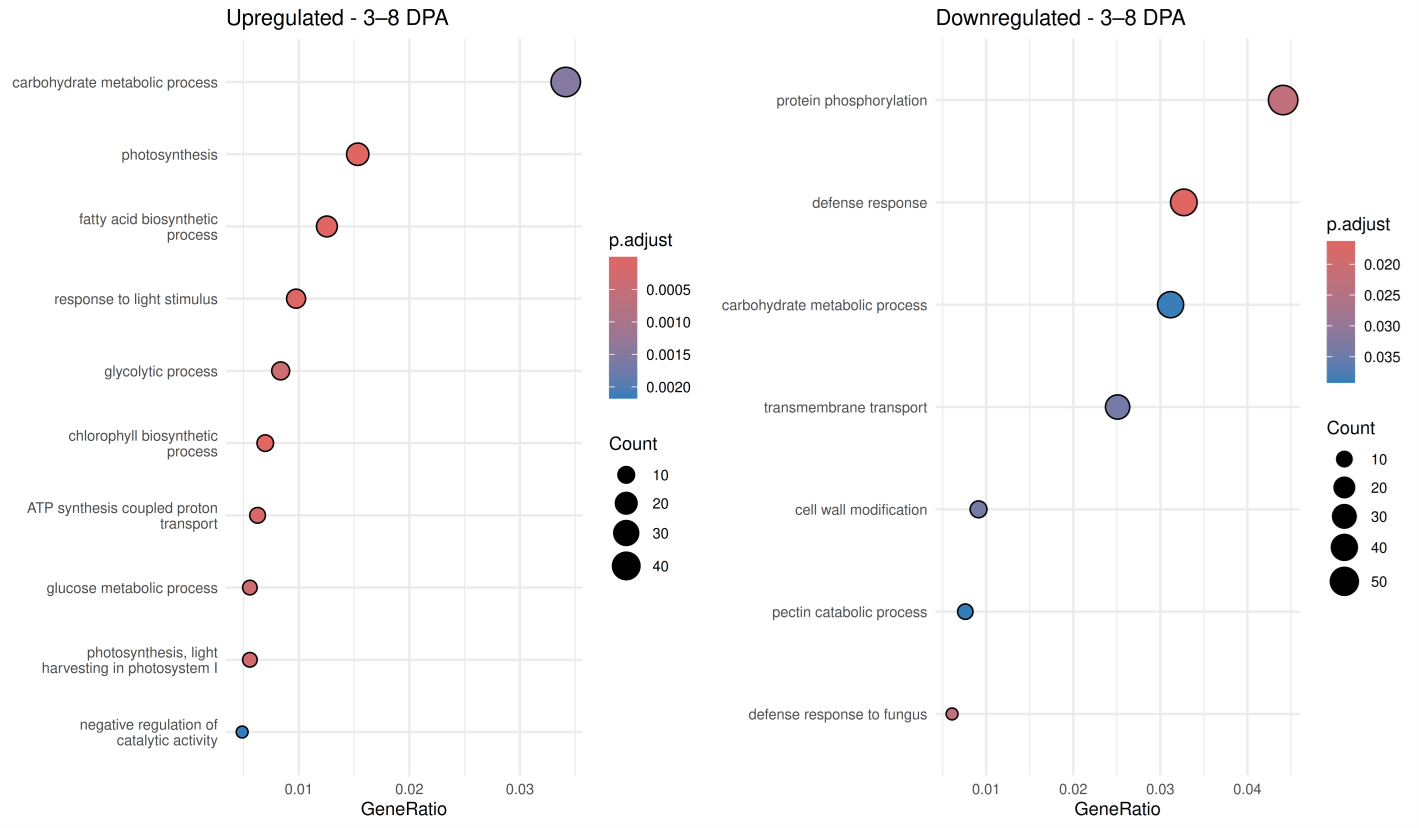

Supplement: Supplementary file 1 [file plants-15-00137-s001.zip › Figure S5.png]

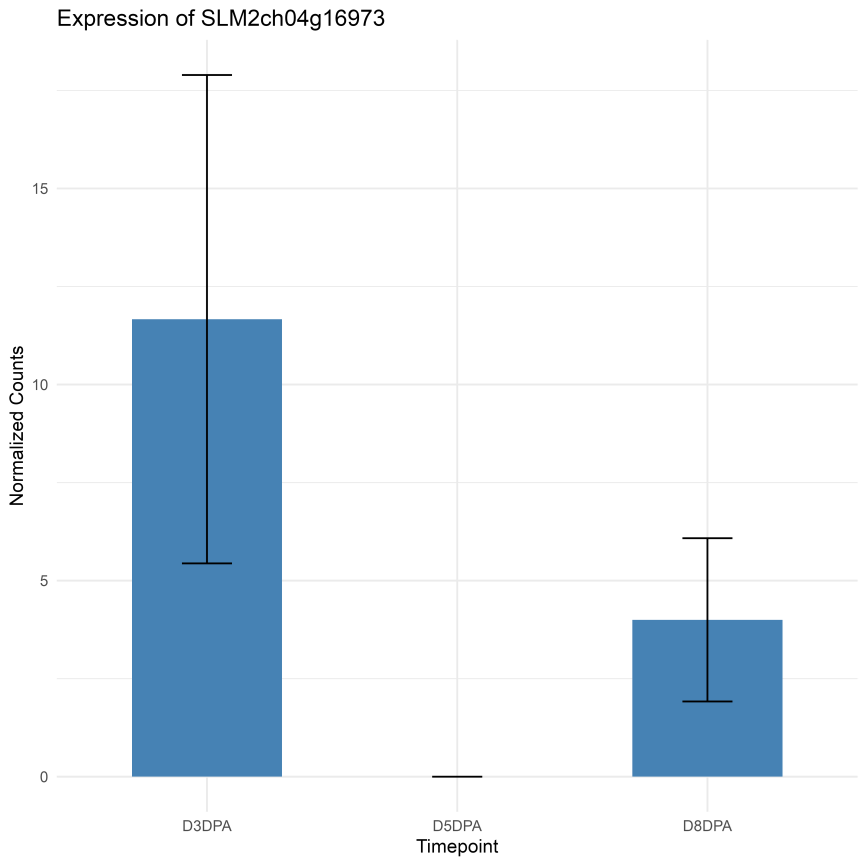

Supplement: Supplementary file 1 [file plants-15-00137-s001.zip › Figure S6.png]

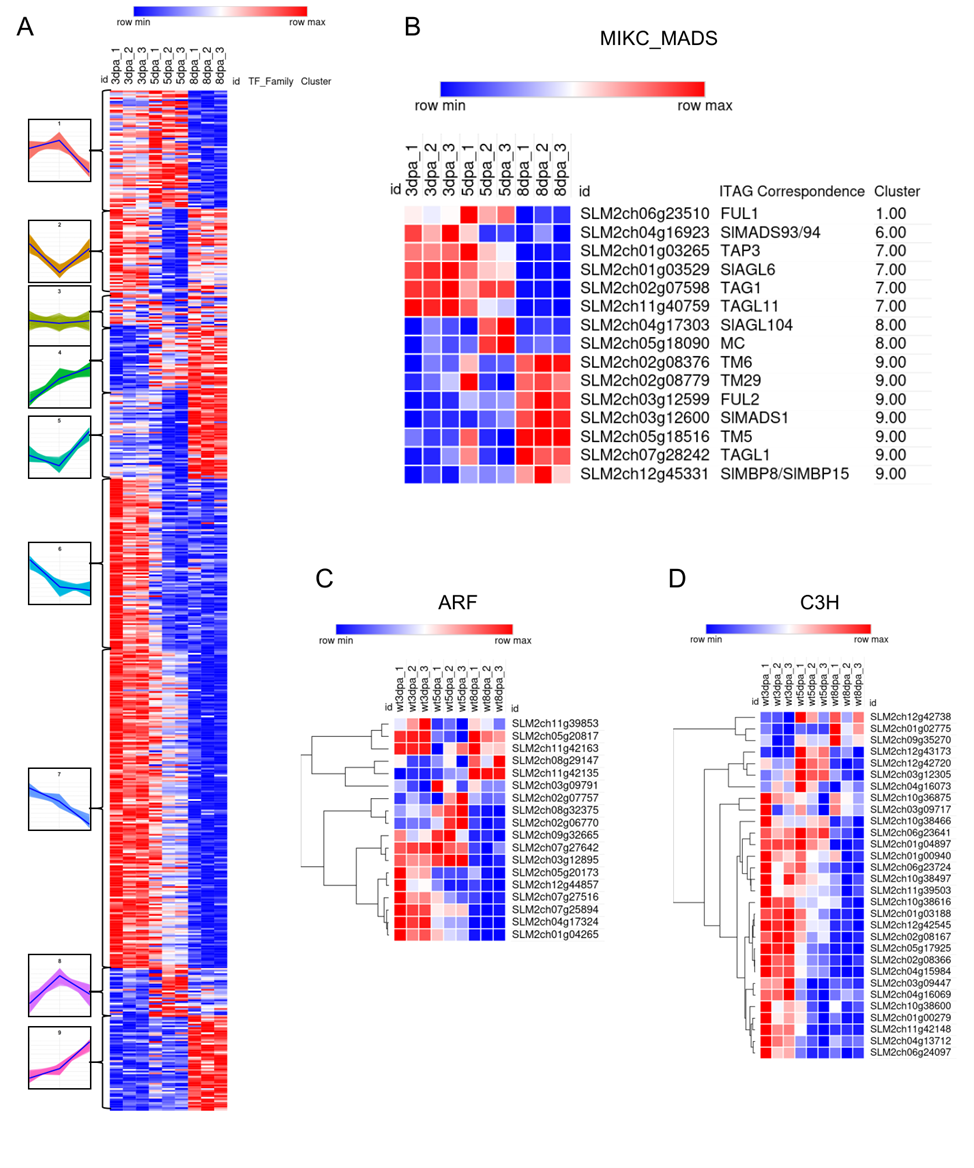

Supplement: Supplementary file 1 [file plants-15-00137-s001.zip › Figure S7.png]
